# The Impact of the Nasal Trauma in Childhood on the Development of the Nose in Future

**DOI:** 10.3889/oamjms.2016.081

**Published:** 2016-08-02

**Authors:** Gabriela Kopacheva-Barsova, Slavica Arsova

**Affiliations:** 1 *University Clinic for Ear, Nose and Throat, Faculty of Medicine, Ss Cyril and Methodius University of Skopje, Vodnjanska 17, Skopje 1109, Republic of Macedonia*; 2 *University Clinic of Psychiatry, Ss Cyril and Methodius University of Skopje, Vodnjanska 17, Skopje 1109, Republic of Macedonia*

**Keywords:** nasal fractures in children, nasal growth, treatment of recent traumatic deformities, rhino surgery in children and adolescents, psychological behaviour

## Abstract

**AIM:**

To prevent and to treat nasal trauma in children properly, because it can lead to displacement or depression of the nasal bones or septum. Second, our aim was, for the patient to recognise and create a mature decision for eventual nose changes which will be made with the operative intervention or they are not mature enough and the decisions were made by their parents.

**MATERIAL AND METHODS:**

Our retrospective study was made at University Clinic for Ear, Nose and Throat, Faculty of Medicine, Ss Cyril and Methodius University of Skopje in the period of 6 years (2005-2016). Seventy-three patients were admitted with recent or previous nasal trauma or nasal deformity. The first group of 32 were children and adolescents from 6-14 years old who were admitted to our hospital because of recent nasal trauma. The second group of 41 children and adolescents from 6-14 years old were admitted to our hospital because of previous nasal trauma, which was not treated on time, or it was not treated properly. They were admitted to our clinic for surgical intervention septo/rhinoplasty. The second group of patients fills the brief psychological questioner prepared by Clinical psychiatrist from University Clinic of Psychiatry, in Skopje, and their psychological reactions were taken into consideration.

**RESULTS:**

Eleven of the children and adolescents who had nasal fracture without dislocation, who have no symptoms, minimal swelling, and no septal deviation or hematoma, were observed with a specific follow-up: 3 days after nasal fracture, then every week in the first month, after 1 month, and after 3 months period. Sixteen of children and adolescents who had a nasal fracture with subluxation of nasal septum were operated with closed reduction (repositio nasi) under general anaesthesia. The others with septal hematomas and subperichondrial abscess were treated as in adults’ patients. The second group of 41 children and adolescents from 6-14 years old consisted with with the previous nasal trauma which was not treated on time or it was improperly treated. In 24 (58.54%) of these patients septoplasty was performed and in 17 (41.46%) was performed rhino septoplasty.

**CONCLUSION:**

Often, difficult septal deformations in children are followed with deformation of the nasal pyramid (rhino scoliosis, rhino lordosis). In those cases, we cannot solve septal pathology without nasal pyramid intervention in the same time and opposite. Clinical reports have not produced solid evidence for the statement that septal surgery has no negative effect on nasal growth or can serve for correcting abnormal growth. The functional and esthetic problems of the patient, however, mean a continuous stimulus for further clinical and experimental investigations.

## Introduction

Nasal fractures have been reported as 1 of the 3 most commonly encountered paediatric facial bone fractures. The nasal bones and mandible are the facial bones most commonly fractured in children. Nasal fractures occur more commonly than do mandibular fractures because they require less force to produce.

The most common causes of nasal fractures in this age group are auto accidents, usually involving bicyclists or pedestrians (40%), sports injuries (25%), intended injuries such as weight lifting (15%), and home injuries (10%). Child abuse also must be considered.

Fractures of the bony nasal pyramid do not occur as frequently as in adults, because of the lesser prominence of the nasal bones during childhood because the greater part of the nasal skeleton is cartilaginous. Therefore, fractures of the facial bones are uncommon occurrences in children younger than five years of age, but the incidence increases with increasing age and peaks between 16 and 20 years. Most of these injuries are minor (almost in 85%) [1-4].

### Anatomy

The sutures bordering the nasal bones have not been yet ossified and can “split off” in a case of trauma. The most frequent lesion is the septal fracture with dislocation. The upper one-third of the nose is supported by the paired nasal bones and the frontal processes of the maxilla; the lower two-thirds are maintained by cartilaginous structures. The superior portion of the nasal bones is thick and relatively resistant to fracture. In contrast, the inferior portion is thin and weak. The bony nasal septum articulates with the undersurface of the nasal bones to provide support to the nasal dorsum. The lacrimal bones and ethmoid labyrinth lie deep to this bony pyramid [5-7].

Blood supply of the nose, derived from the internal and external carotid arteries. Kiesselbach plexus, the vascular watershed area of the anteroinferior nasal septum, is the site of origin of most nosebleeds. Because of the rich nose blood supply frequency and intensity of epistaxis and swelling in patients with nasal trauma are very common.

### Pathogenesis

Direct blows to the nose can fracture the nasal skeleton and lead to displacement or depression of the nasal bones or septum. Because the nasal pyramid is variably cartilaginous during childhood, greenstick fractures are more common.

Rupture of a triangular (dorsolateral) cartilage from the piriform aperture is a difficult diagnosis. It appears with dorsum hematoma, caused by rupture of the external brunch of the anterior ethmoid artery, On the outside, the hematoma is often connected with facial oedema. Expansion of the hematoma separates the cartilage from the mucoperichondrium, obstructing blood flow to the nasal cartilage and causing pressure-induced avascular necrosis of the nasal cartilage. Septal perforation and irreversible damage result after three to four days of ischemia. The accumulated blood and necrotic tissue become a “good” place for infection of the nasal mucosa [8, 9].

The replacement of necrotic tissue by fibrous tissue, retraction of scar tissue, and loss of support to the lower nose may lead to facial deformity, including saddle nose, displacement of the maxilla, retraction of the anterior nasal septum (columella), widening of the nasal base, and diminished size of the nasal cavity [10, 11].

### Naso-orbito-ethmoid fractures

Naso-orbito-ethmoid fractures occur with high impact to the central midface and involve complete separation of the nasal bones and the medial walls of the orbits from the frontal bone and the infraorbital rim. The bones typically are fragmented and displaced posteriorly into the ethmoid region. The medial canthal tendons, which are attached to the medial walls of the orbits, are shifted laterally with the fracture segments, resulting in increased inner canthal distance (traumatic telecanthus) [12, 13].

### Clinical manifestation of nasal fractures in children

**Figure 1:**
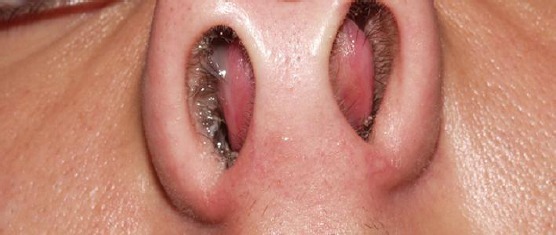
Septal hematoma after previous nasal trauma

Unfortunately, most of the nasal fractures in children are not refers to the ENT - specialist immediately. Evaluation of nasal symptoms following nasal trauma includes nasal obstruction, nasal bleeding, pain, anosmia, and cosmetic deformity. The typical symptoms of nasal septal hematoma and/or abscess are a progressive nasal obstruction (95 percent), persistent or worsening pain (50 percent), rhinorrhea (25 percent). All children with facial trauma should be assessed for associated injuries to the cervical spine, central nervous system, chest, orbits, and teeth [14-16] (Fig. 1).

If symptoms present with the change in visual acuity, diplopia, and sensory deficits should have a prompt evaluation for neurologic or ophthalmologic trauma.

### Patient physical examination and evaluation

The septum should be examined for fracture, displacement, laceration, discoloration, and abnormal swelling. The intranasal cavity should be evaluated using an appropriate lighting in all children with nasal trauma. The signs and symptoms of nasal septal injury may evolve during the 24 to 72 hours after injury.

A correct diagnosis of fracture and dislocation of the bony and cartilaginous nasal pyramid is more difficult in children than in adults, because of the smaller dimensions and abundant swelling which is very often due to oedema and or hematoma and less cooperation of the patient. The examination should be repeated after two or three days, when the soft tissue swelling has diminished [17, 18].

The examination of children with nasal trauma should include inspection of the nose and facial structures, palpation of the facial and nasal bones, and internal examination of the nose. Periorbital ecchymoses in the absence of other orbital findings are suggestive of a nasal fracture.

External nasal deformity, epistaxis, oedema, and ecchymosis suggest septal injury. A flattened, broad nose with increased inner canthal distance and vertical orbital displacement suggest a nasal-orbito-ethmoid fracture.

Tenderness, deformity, mobility, crepitus, or step should be evident on the nose palpation. Tenderness over the frontal sinus may indicate frontal sinus fractures. Tenderness to palpation of the tip of the nose is suggestive of septal hematoma. Malocclusion and instability of the palate are indicative of a midfacial LeFort fracture.

In addition, nasal bleeding and swelling, which occur soon after trauma, can impair the evaluation [19-21]. Applying local pressure or topical vasoconstricting agents (e.g., phenylephrine nasal spray, two drops in each nostril) or nasal packing may be necessary to improve the ability to assess the septum for hematoma or deviation.

### Imaging Evaluation

Modern computed tomography (CT) is the gold standard for viewing craniofacial and nose fractures. CT images provide excellent detail of the cranium, midfacial structures, and the mandibular condyle. In addition to sagittal and coronal views, reformatting images into a three-dimensional reconstruction provides an improved perspective in complex injuries.

**Figure 2:**
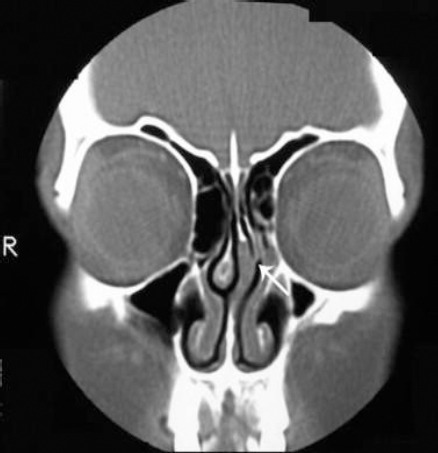
CT tomography of nasal fracture

Plain radiographs are not helpful in the diagnosis of nasal fractures in children and should not be substituted for a complete external and internal examination. The nasal bones of children are poorly visualised on plain radiography because they are not fused and are composed primarily of cartilage [22, 23] (Fig. 2).

## Material and Methods

In our retrospective study which was made at University Clinic for Ear, Nose and Throat, Faculty of Medicine, Ss Cyril and Methodius University of Skopje in the period of 6 years (2005-2016), Seventy-three patients were admitted with recent or previous nasal trauma or nasal deformity.

The first group of 32 were children and adolescents from 6-14 years old who were admitted to our hospital because of recent nasal trauma (nasal bone fracture, or together with consecutive complications caused by recent nasal trauma) were observed. All of them were examined by rhinoscopy, plain radiography or CT scan of nose and paranasal sinuses depending on the severity of the nasal fracture.

The second group of 41 children and adolescents from 6-14 years old were admitted to our hospital because of previous nasal trauma, which was not treated on time, or it was not treated properly. They were admitted to our clinic for surgical intervention septo/rhinoplasty. They had the first nasal trauma in early childhood or several years before they came for surgery.

## Results

**Figure 3:**
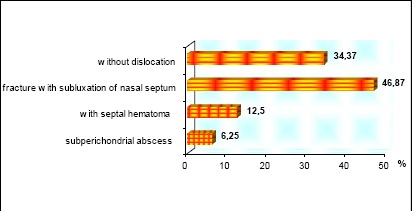
Distribution of recent nasal fracture and associated symptoms in children and adolescents

The first group consisted of 32 children and adolescents, 11 were with nasal fracture without dislocation, 15 with nasal fracture with septal subluxation, in 4 of the children septal hematoma were observed and in 2 cases subperichondrial abscess appeared (Fig. 3).

Eleven of the children and adolescents who had nasal fracture without dislocation, who have no symptoms, minimal swelling, and no septal deviation or hematoma, were observed with a specific follow-up: 3 days after nasal fracture, then every week in the first month, after 1 month, and after 3 months period. Fortunately, after the observation, surgery, reposition on the nose bones or septoplasty was not performed on any of them.

Sixteen of children and adolescents who had a nasal fracture with subluxation of nasal septum were operated with closed reduction (repositio nasi) under general anaesthesia. The others with septal hematomas and subperichondrial abscess were treated as in adults’ patients (Table 1).

**Table 1:** Treatment of recent nasal trauma in children in adolescents

Treatment of recent nasal trauma in children in adolescents		N (%)
Nasal fracture without dislocation	Without intervention	11 (34.37)
Nasal fracture with subluxation	Repositio nasi	15 (46.87)
Septal hematoma	Conservative treatment	4 (12.5)
Septal abscess	Conservative treatment	2 (6.25)

The second group of 41 children and adolescents from 6-14 years old were admitted to our hospital because of previous nasal trauma which was not treated on time or it was improperly treated. They were admitted to our clinic for surgery septo/rhinoplasty. They had the first nasal trauma in early childhood or several years before they came for surgery. In 24 (58.54%) of these patients septoplasty was performed and in 17 (41.46%) was performed rhino septoplasty.

The second group of patients fills the brief psychological questioner prepared by Clinical psychiatrist from University Clinic of Psychiatry, University Campus “St. Mother Theresa” in Skopje, and their psychological reactions were taken into consideration. The aim was, for the patient to recognise and create a mature decision for eventual nose changes which will be made with the operative intervention or they are not mature enough and the decisions were made by their parents. We ask them whether the surgery will change their emotional and psychological way of living.

About their self-concerning, the result shows that most of them 63.9% were middle concerned, dominant of them, 75%, have real expectations from the operation. Their Answer to the question “Why you are doing this operation”, 24.39% thought that the intervention will make to look aesthetically better, both (51.22%) the breathing will be better too (Table 2).

**Table 2:** Patients self-concerning for their nasal deformities

Variable	N (%)
***How big is their self-concern?***	
Little concern	6 (14.63)
Middle concern	26 (63.41)
Very concern	9 (21.95)
***Their expectations?***	
Real	31 (75.61)
Unreal	7 (17.07)
Impossible	3 (7.32)
***Why is the operation important for them?***	
Aesthetic moment	10 (24.39)
Breathing disturbances	11 (26.83)
Both	21 (51.22)

**Figure 4:**
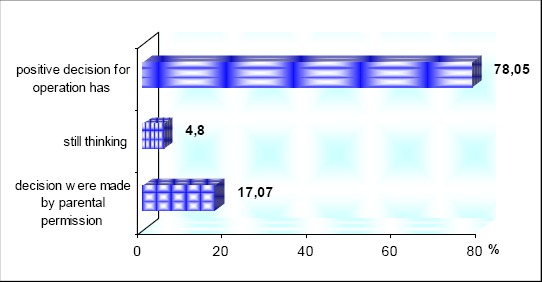
Psychological maturity and their decision for operation

During the testing, their psychological maturity and their decision for the operation were observed. Positive decision for operation has 32 (78.05 %) of course with parental permission, 2 (4.8%) of them were still thinking even their parents allowed them, and in 7 (17.07%) decision for rhino/septoplasty were made by parental permission and opinion because they were not mature enough to conclude the right decision (Fig. 4).

### Case reports

**Figure 5:**
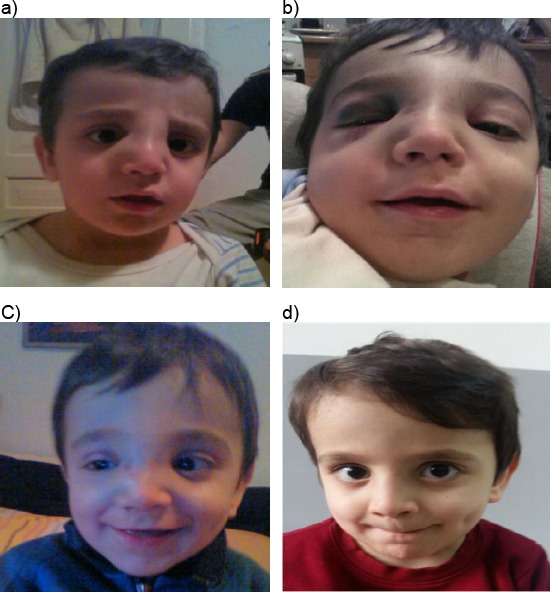
Four years old boy with nasal fracture without dislocation caused by hitting his head on a table. a) 2 days after the injury; b) 7 days after the injury; c) 14 days after the injury; d) 1 month after the injury (with the permission of parents)

Case 1: Four years old boy with nasal fracture without dislocation caused by hitting his head on a table (Fig. 5).

**Figure 6:**
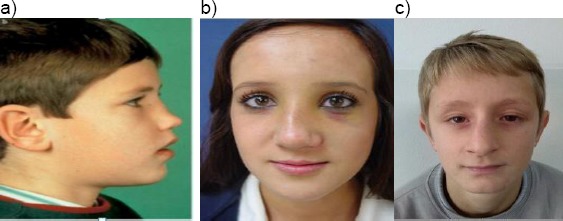
a) Six and a half-year-old boy with saddle deformity after septum abscess at the age of 4 years; b) Twelve years old girl with nasal septal subluxation after a fall. Five days after injury; c) Ten years old boy with nasal septal subluxation after a fall. Three months after injury (with the permission of parents)

Case 2: a) Six and a half-year-old boy with saddle deformity after septum abscess at the age of 4 years; b) Twelve years old girl with nasal septal subluxation after a fall. Five days after injury; c) Ten years old boy with nasal septal subluxation after a fall. Three months after injury (Fig. 6).

**Figure 7:**
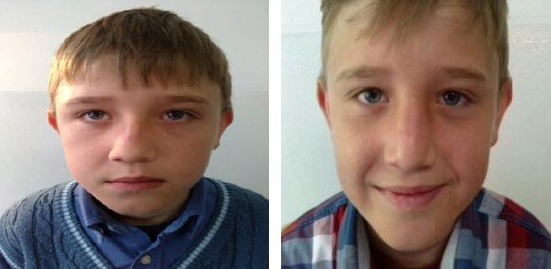
Eight years old boy with nasal septal subluxation after a fall. a) Three days after injury; b) Seven days after reposition (with the permission of parents)

Case 3: Eight years old boy with nasal septal subluxation after a fall (Fig. 7).

**Figure 8:**
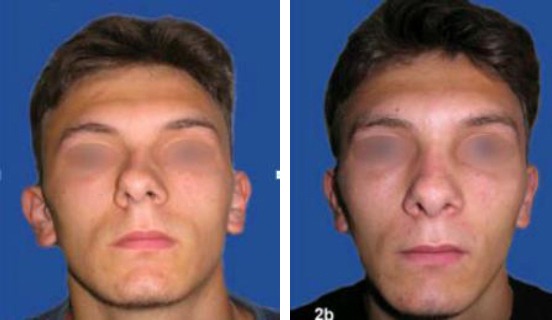
Fourteen years old boy with subluxation of nasal septum after nasal trauma in a previous car accident. a) Preoperative results; b) post-operative results

Case 4: Fourteen years old boy with subluxation of nasal septum after nasal trauma in a previous car accident. Septoplasty was prepared. Septal media position (Fig. 8).

**Figure 9:**
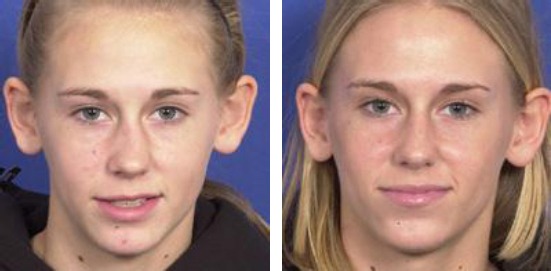
Fourteen years old girl with rhino scoliosis after nasal trauma in early childhood. a) Preoperative results; b) postoperative results (with the permission of parents)

Case 5: Fourteen years old girl with rhino scoliosis after nasal trauma in early childhood. Septo/rhinoplasty with closed approach was performed. Medial and lateral osteotomy. Septal media position (Fig. 9).

**Figure 10:**
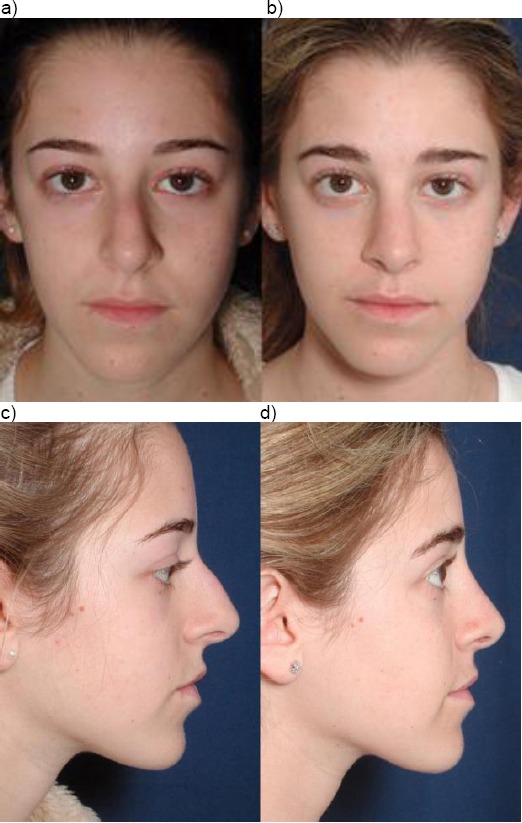
Twelve years old girl with rhino kyphosis after nasal trauma in early childhood. a) Preoperative; b) postoperative; c) pre-operative; d) postoperative (with the permission of parents)

Case 6: Twelve years old girl with rhino kyphosis after nasal trauma in early childhood. Septo/rhinoplasty with closed approach was performed. Medial and lateral osteotomy. Septal media position. Hump reduction (Fig. 10).

**Figure 11:**
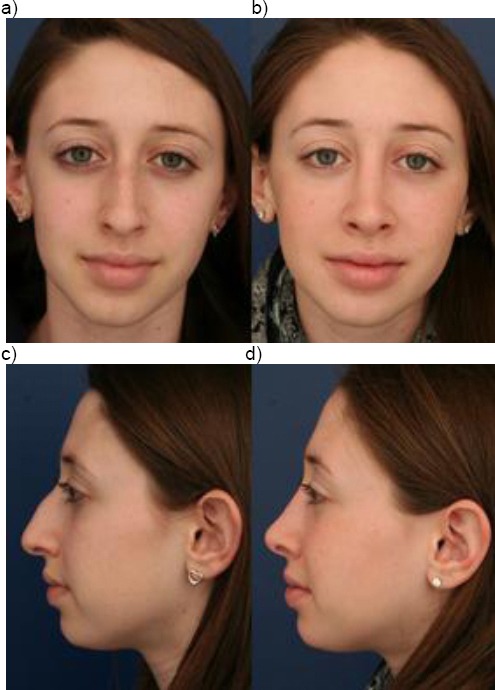
Fourteen years old girl with rhino kyphosis after nasal trauma in early childhood. a) Preoperative; b) postoperative; c) pre-operative; d) postoperative (with the permission of parents)

Case 7: Fourteen years old girl with rhino kyphosis after nasal trauma in early childhood. Septo/rhinoplasty with closed approach was performed. Medial and lateral osteotomy. Septal media position. Hump reduction (Fig. 11).

## Discussion

The management of nasal trauma in children and adolescents depends on upon their age, the degree of nasal obstruction, and associated injuries. A correct diagnosis of the fracture of the nasal pyramid (bony or cartilaginous) is more difficult in children than in adults. Patients who have no symptoms, minimal swelling, and no septal deviation or hematoma do not need specific follow-up. Patients with a nasal fracture but no septal hematoma may be referred to the otorhinolaryngologist within three to five days. The examination should be repeated after 3 days and after one week when the soft tissue swelling has diminished. Most nasal fractures in children can be managed with closed reduction under general anaesthesia. Fractures with splayed nasal bones and no impactions can be reduced with bilateral digital compression on the dorsum for 10 to 15 minutes. Use of intranasal instrumentation may be necessary if digital compression alone is not successful.

Open reduction may need to be performed if significant dislocations are present; the injury is more than two weeks old, or closed and intranasal instrumentation fail. In exceptional cases, it is necessary to use a 2mm osteotome to produce a satisfactory alignment of the nasal bones. Children with septal hematomas or abscesses warrant prompt paediatric otolaryngology consultation. Septal hematoma and abscesses are treated with incision, drainage, and nasal packing. Nasal packing for a few days is only tolerated by older children. In addition, if parents are instructed to bring a recent photograph of the child to the appointment, the otorhinolaryngologist can compare the nasal contours before and after the trauma [24-26].

After the nasal bones reposition, the nasal bones remain mobile for approximately two weeks and can be depressed by force for up to six weeks. Thus, recommendation for the children and the adolescents are to avoid all sports activities for two weeks and some contact sports (karate, wrestling) for six weeks. Most athletes successfully return to their sport after a nasal fracture. However, if these nasal fractures are not treated in time, or if they are not treated at all, they can cause difficult septal deformations, followed by deformation of the nasal pyramid (rhino scoliosis, rhino lordosis, rhino kyphosis) later in life. This happens because the childhood trauma caused a negative effect on nasal growth and serious damage on the nasal growth spurts and the development and ossification of the nose. These deformities can later cause changes in the emotional development of the young adolescents, making them the next candidates for septal/rhinoplasty [27, 28].

Nasal trauma in children can fracture the nasal skeleton and lead to displacement or depression of the nasal bones or septum. Because the nasal pyramid is variable cartilaginous during childhood, and the midline suture is no fused, greenstick fractures are more common. Evaluation of nasal symptoms following nasal trauma includes assessment of nasal obstruction, nasal bleeding, pain, anosmia, and cosmetic deformity. Symptoms of nasal septal hematoma and/or abscess consist of progressive nasal obstruction, persistent or worsening pain after trauma, rhinorrhea, and fever. The clinician should perform a complete external and internal examination of the nose with attention to signs of associated injuries, including cervical spine injury, cerebrospinal fluid leak, naso-orbito-ethmoid fractures, and LeFort fractures. Plain radiographs are not helpful in the diagnosis of nasal fractures in children and should not be performed solely for this purpose. Nasal fractures with a deviation of the nasal septum should be reduced by an otorhinolaryngologist within seven days.

With advances in prevention, imaging evaluation, and bone fixation technology, the management of paediatric facial fractures continues to evolve at a rapid pace. Although often complex, effective management of fractures within this challenging population is directly dependent upon thorough initial evaluation, correct injury assessment, and timely initiation of chosen therapy. Although facial fractures in this group are uncommon relative to their adult counterparts, a thorough understanding of issues relevant to paediatric facial fractures is critical in providing ideal acute management and optimising long-term success [29-31].

In summary, the growth centres of the nose have to be avoided if possible; long-term nasal issues will theoretically be minimised. If the surgeon replaces it, the cartilage of the nose becomes straighter but still intact.
